# Curved Beam Computed Tomography based Structural Rigidity Analysis of Bones with Simulated Lytic Defect: A Comparative Study with Finite Element Analysis

**DOI:** 10.1038/srep32397

**Published:** 2016-09-02

**Authors:** R. Oftadeh, Z. Karimi, J. Villa-Camacho, E. Tanck, N. Verdonschot, R. Goebel, B. D. Snyder, H. N. Hashemi, A. Vaziri, A. Nazarian

**Affiliations:** 1 Center for Advanced Orthopaedic Studies, Department of Orthopaedic Surgery, Beth Israel Deaconess Medical Center, Harvard Medical School, Boston, MA, USA; 2 Department of Mechanical and Industrial Engineering, Northeastern University, Boston, MA, USA; 3 Orthopaedic Research Laboratory, Radboud University Medical Center, Nijmegen, the Netherlands; 4 Sport Science Program, Qatar University, Doha 2713, Qatar

## Abstract

In this paper, a CT based structural rigidity analysis (CTRA) method that incorporates bone intrinsic local curvature is introduced to assess the compressive failure load of human femur with simulated lytic defects. The proposed CTRA is based on a three dimensional curved beam theory to obtain critical stresses within the human femur model. To test the proposed method, ten human cadaveric femurs with and without simulated defects were mechanically tested under axial compression to failure. Quantitative computed tomography images were acquired from the samples, and CTRA and finite element analysis were performed to obtain the failure load as well as rigidities in both straight and curved cross sections. Experimental results were compared to the results obtained from FEA and CTRA. The failure loads predicated by curved beam CTRA and FEA are in agreement with experimental results. The results also show that the proposed method is an efficient and reliable method to find both the location and magnitude of failure load. Moreover, the results show that the proposed curved CTRA outperforms the regular straight beam CTRA, which ignores the bone intrinsic curvature and can be used as a useful tool in clinical practices.

The skeleton is the third most common site of metastatic cancer, and nearly half of all cancers metastasize to bone^1^,^2^,^3^. Approximately 30–50% of bone metastases lead to pathologic fractures^4^, where the orthopaedic surgeon faces the dilemma of determining the probability of such an event, oftentimes based on subjective assessments of bone strength. Patients deemed to have a low risk of fracture are treated using nonsurgical approaches^5^,^6^, and operative treatment is reserved for cases of impending and pathological fractures in long bone and pelvic girdle metastases. Nevertheless, the scoring systems frequently used to evaluate fracture risk are now recognized to be inaccurate^7^. Therefore, there is a need for a reliable clinical tool to objectively assess fracture risk based on the material and geometric determinants of bone strength.

Computed tomography-based structural rigidity analysis (CTRA), which takes into account the material properties and structural organization of bone, can reliably predict failure load in rat and human bones with lytic defects^8^,^9^,^10^,^11^,^12^,^13^,^14^. However, CTRA calculations are derived from straight beam theory, where the influence of bone curvature on strength has not been considered . This influence has been shown to be significant in calculating bone fracture load^15^. Therefore, in current study, a new method for evaluating CTRA based on curved beam theory (curved CTRA) has been introduced. To that end, failure load predictions from curved CTRA, traditional CTRA and Finite Element (FE) modeling has been compared to those of mechanical testing in an *ex-vivo* human model of femoral lytic defects. We hypothesize that curved beam CTRA will outperform traditional CTRA in terms of the accuracy of the predicated failure load and also the failure location and will correlate well with FEA and mechanical testing results.

## Material and Methods

### Specimen Preparation

Following Institutional Review Board approval and in accordance with guidelines for use of cadaveric specimens with informed consents obtained, ten paired femurs from fresh frozen human cadavers (mean age 81.7 ± 10.65 years) were obtained from the Department of Anatomy at Radboud University Medical Center^16^,^17^. One of the femurs in each pair was left intact and assigned to the control group. The contralateral femur was assigned to the simulated lytic defect group, where one or more defects were created. Size and location of these lesions resembled clinical appearance of lytic metastatic lesions, as discussed with orthopedic oncologists. Lesion sizes and locations on defect femurs are shown in Table 1.

### Imaging and Image Analysis

Quantitative computed tomography (QCT) images were acquired with the following settings: 120 kVp, 220 mA, slice thickness 3 mm, pitch 1.5, spiral and standard reconstruction, in-plane resolution 0.9375 mm (ACQSim, Philips, Eindhoven, The Netherlands)^16^. The femurs were scanned in a water basin, on top of a solid calibration phantom (Image Analysis, Columbia, KY, USA).

### Mechanical Testing

**Figure 1:**
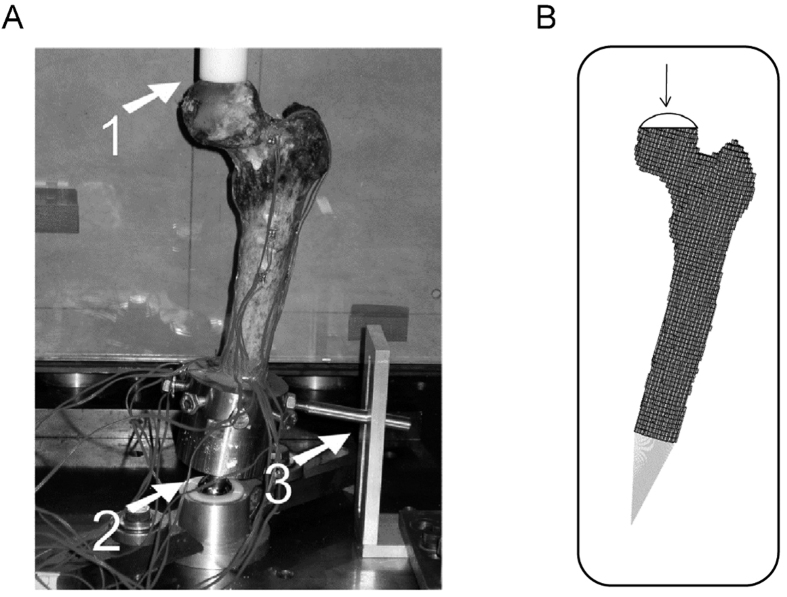
Mechanical testing setup (1) the load is applied with plastic cup (2) the point of rotation and (3) All rotations are restricted except the rotation around the AP-axis^16^. (**B**) Representing finite element model.

Following imaging, the specimens underwent mechanical testing in a hydraulic mechanical testing system (MTS, Eden Prairie, MN, USA)^16^. The setup was designed to simulate single-limb stance-type loading conditions on the femur (Fig. 1A). A 30 mm diameter plastic cup with concave shape was used to apply the load to the femoral head. An axial load was applied on the head of the femur, with 10 N/s from 0 N until failure, while force and displacement of the plunger were recorded. The failure location of each femur was photographically documented.

### Finite Element Analysis

Three dimensional (3D) models of the femurs were constructed using MATLAB (MathWorks Inc., Natick, MA, USA). The calcium-phosphate (Ca-P) density (ρ_CHA_) of each pixel in the CT scan was calculated using the calibrating phantoms^18^ (the pixels below 30 mg/ml were automatically removed), and a 3D model was constructed using mutually connected pixels^19^,^20^. Using the relationship *ρ*_*ash*_ = 0.0633 + 0.887*ρ*_*CHA*_^21^, the Ca-P densities were transformed to bone mineral density (*ρ*_*ash*_). Then, the mineral densities were converted to tissue elastic modulus using empirically derived constitutive equations for cancellous^22^,^23^ and cortical^24^ bone. Elastic-perfectly plastic isotropic material behavior was assigned to model the mechanical behavior of bone material, and constrains and boundary conditions were applied to the model to mimic the mechanical testing experiments^16^. The developed models were imported to finite element (FE) software, ANSYS (Academic Research, Release 14.0, Cecil, PA, USA), which was used to perform the FE analysis. Each model was rotated to the corresponding orientation of the femur in mechanical testing based on the position of the tantalum markers in the CT scans. Finite element analysis was conducted in displacement control, and the displacement was applied incrementally using a cup with 30 mm diameter consistent with the mechanical tests (Fig. 1B). The distal femur was fixed using two bundles of high-stiffness springs to imitate the experiments (Fig. 1B). Plastic behavior was excluded from the elements in the top surface in contact with cup to prevent unrealistic distortion. For each displacement increment (i.e. 0.025 mm), the total reaction force was calculated as the sum of all nodal forces in contact with the cup. The failure load in FE analysis was defined as the maximum total reaction force achieved during loading under displacement control. Failure location in the computational models was defined by the location of maximum effective plastic strain at the maximum total reaction force.

### Structural Rigidity Analysis

**Figure 2:**
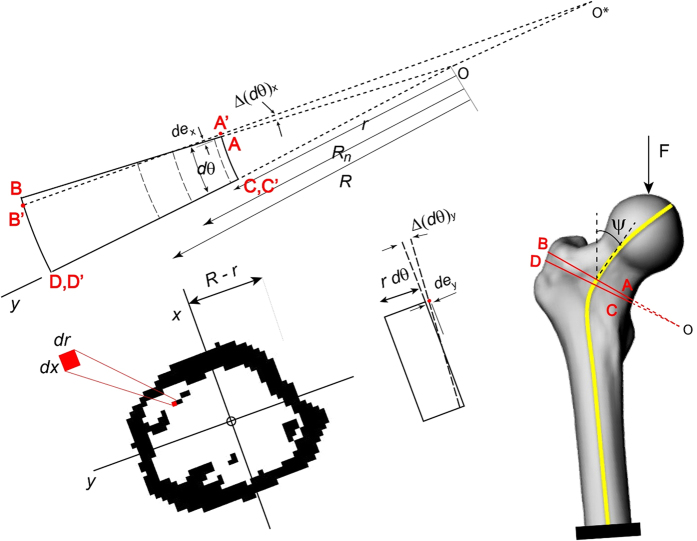
Representation of the curve beam model. (ABCD section from right figure is shown on the left before and after deformation with corresponding strains).

CTRA determines bone rigidity and likelihood of failure based on the axial (EA), bending (EI), and torsional (GJ) rigidities of the weakest cross-section in the bone^8^. These parameters were evaluated at each transaxial cross-section by summing the rigidity of all pixels (Fig. 2).

The failure load of femoral bone subject to axial and bending moment can be obtained by assuming that planes stay plane in deformation, and shear deformation is neglected. For a general asymmetric cross section and assuming straight beam, the failure load can be expressed as:


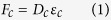


where *D*_*c*_ is the rigidity at the weakest bone cross-section and *ε*_*c*_ is the critical strain which identifies the fracture initiation. Using straight beam theory^25^, *D*_*c*_ can be defined as:


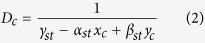


where *γ*_*st*_ is associated with axial rigidity, and *α*_*st*_ and *β*_*st*_ are associated with bending rigidity of the cross section. *x*_*c*_ and *y*_*c*_ are coordinates of critical location in the weakest cross-section. *γ*_*st*_, *α*_*st*_ and *β* are defined as:


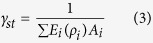



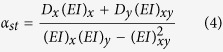



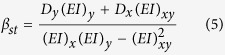


where *D*_*x*_ and *D*_*y*_ are the distances, from the geometric centroid, to the applied load in x and y directions. *E*_*i*_ and *ρ*_*i*_ are the elastic modulus and density, respectively, at the *i*th location of the cross-section, and *A*_*i*_ is the incremental cross-sectional area (Fig. 2) and (*EI*)_*x*_, (*EI*)_*y*_ and (*EI*)_*xy*_ correspond to the bending rigidities of the cross-section respect to its centroid and can be defined as:


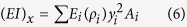



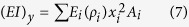



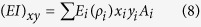


The centroid of cross section is obtained from


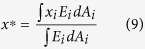



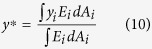


As the traditional CTRA is based on straight beam theory, its main disadvantage is that it does not account for the influence of intrinsic bone curvature. This influence is particularly important for structures, where the ratio of the radius of curvature to the depth of a beam is less than 5^26^, and neglecting curvature effect can cause a meaningful underestimation of resulting stresses^25^. The intertrochanteric region of the human femur has the highest curvature, this ratio is around 1^27^. Therefore, in this case, it is essential to account for bone curvature in CTRA analyses. Consider the sample human femur bone cross-section shown in Fig. 2. The curved cross sections are found based on iteratively find the tangent vectors of curve passing through the centroid of cross sections and perpendicular to them. The iteration stops when there is no change on the position of tangent vectors and subsequently the position of curved cross sections. Circumferential stress *σ*_*θθ*_ on the ABCD section can be found by balancing the resultant forces and moments (*N*, *M*_*x*_ and *M*_*y*_) acting on the cross-section with circumferential stresses. Shear stress *σ*_*rθ*_ can be neglected compared to *σ*_*θθ*_ for thick cross sections^25^:


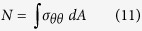



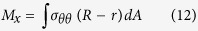



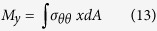


*r* is the distance of the center of curvature from an infinitesimal area (*dA*), and *R* is the distance of the center of curvature to the centroid of the whole cross-section. In Fig. 2, *A*′*B*′*C*′*D*′ is a deformed shape of a curved element *ABCD.* The relative movement of typical point in *yz* plane due to deformation (*de*_*x*_) is equal to (*R*_*n*_ − *r*)Δ(*dθ*)_*x*_, where *R*_*n*_ neutral axis radius and Δ(*dθ*)_*x*_ is the angle between the deformed surface and the original surface in *yz* plane. The relative movement of the point in *xz* plane due to deformation (*de*_*y*_) is equal to *x*Δ(*dθ*)_*y*_, where Δ(*dθ*)_*y*_ is the angle between the deformed surface and the original surface in *xz* plane. Therefore, the total normal strain can be evaluated as:


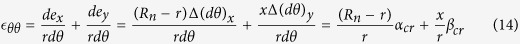


The resultant force and moments can be written as:


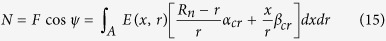



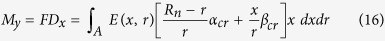



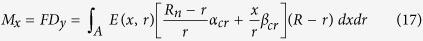


where *F* is the external force applied to the bone, *D*_*x*_ and *D*_*y*_ are the distances of applied load to the centroid axes of the weakest cross-section, and *ψ* is the angle between the external force and resultant normal force on the cross-section. Equations (15) to (17), 16, 17 should be solved simultaneously to find *α*_*cr*_, *β*_*cr*_ and *R*_*n*_, which *R*_*n*_ is the neutral axis radius. In the most general case *α*_*cr*_, *β*_*cr*_ and *R*_*n*_ can be found as:


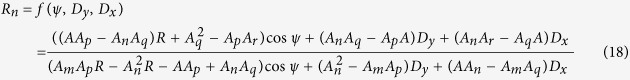



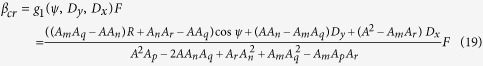



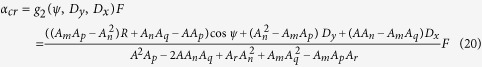


where *R* is the radius of curvature, 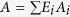, 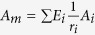, 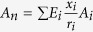, 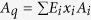, 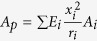 and 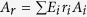. If *r* and *x* are calculated from the modulus weighted surface centroid (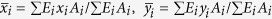), then 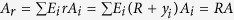 and *A*_*q*_ = 0, then equations (18) to (20) are reduced to:


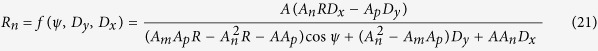



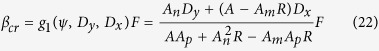



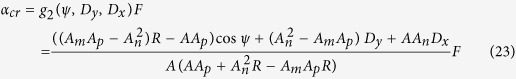


Therefore, critical force can be found as:


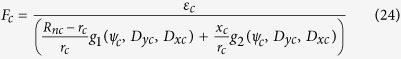


where subscript *c* indicates the parameters values at the weakest location on the bone. The critical strain, where fracture is eminent, is set to 1.2% strain in compression, and 1% strain in tension^11^,^28^,^29^,^30^.

### An Ideal case

**Figure 3:**
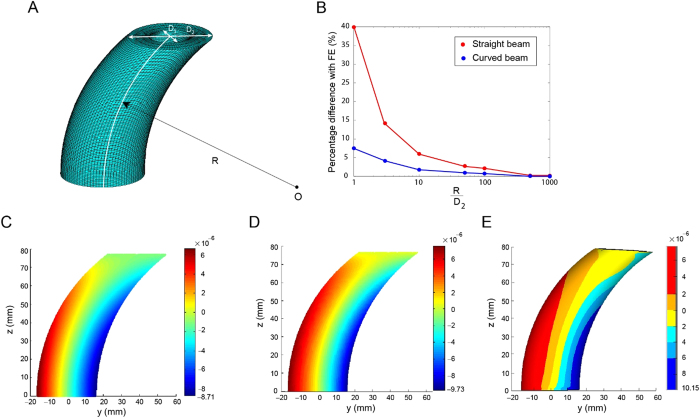
Ideal case (**A**) schematic representation of hallow curved shaft used in ideal case example with radius of curvature *R*, inner diameter *D*_1_ and outer diameter *D*_2_. (**B**) Percentage difference of critical strain in curved beam and straight beam model from that of finite element for various *R*/*D*_2_ (**C–E**) contour plot of maximum principal strain for hollow curved beam *R*/*D*_2_ = 3 based on straight beam (left figure), curved beam (middle figure) and finite element analysis (right figure).

The capability of the proposed model was examined by considering an ideal case of a human femur. The results of straight beam and curved beam models were compared with results from finite element analysis. To simulate the bone femur shape, a hollow shaft with inside diameter (*D*_1_) of 16 mm and outside diameter (*D*_2_) of 32 mm was constructed. The finite element model was also constructed using ANSYS (Academic Research, Release 14.0, Cecil, PA, USA). The ratio of the radius of curvature to outside diameter (*R*/*D*_2_) was changed from 3 to 1000 in the three models to determine the effect of curvature on the maximum principal strain at the critical location. A nominal pressure of 1 Pa was applied at the top surface, while the bottom surface was fully restrained (Fig. 3A).

## Results

Schematic representation of hallow curved shaft used in the ideal case is shown in Fig. 3A. The results for the ideal case are shown in Fig. 3B, based on the difference of each model from FE analysis for the maximum principal strain. For small *R*/*D*_2_ ratios, the straight beam model is significantly biased, while the curved beam model exhibits a better correlation. As *R*/*D*_2_ increases, the two models converge. The strain contour plots for *R*/*D*_2_ = 3 are shown in Fig. 3C–E. Again, the curved beam model demonstrates a better correlation to FEA than the straight beam model.

**Figure 4:**
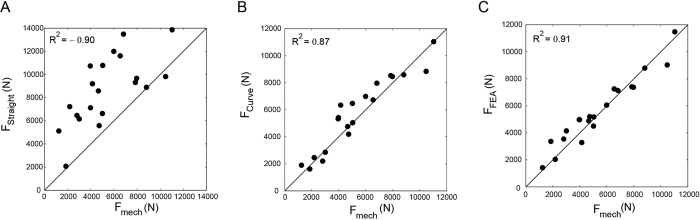
Linear regression between failure loads predicted by (**A**) straight beam model (**B**) curved beam model and (**C**) Finite element analysis versus failure load from mechanical testing.

Lesion sizes and locations on defect femurs are shown in Table 1. Mechanical testing setup, representing finite element model and Representation of the curve beam model are shown in Figs 1 and 2 respectively. The FE and curved beam CTRA models predicted the failure loads for intact femurs and femurs with lytic defects and exhibited a strong level of correlation with mechanical testing results (*R*^2^ = 0.91 and 0.87 against the line of equality, respectively; Fig. 4). On the other hand, the straight beam CTRA model overestimated the failure load in almost all cases and predicts larger failure loads than those reported from experiments. The regression analysis gives the negative coefficient of determination of −0.9 against the line of equality which shows the line of equality does not follow the trend between the failure load from mechanical testing (*F*_*mech*_) and straight CTRA (*F*_*Straight*_).

**Figure 5:**
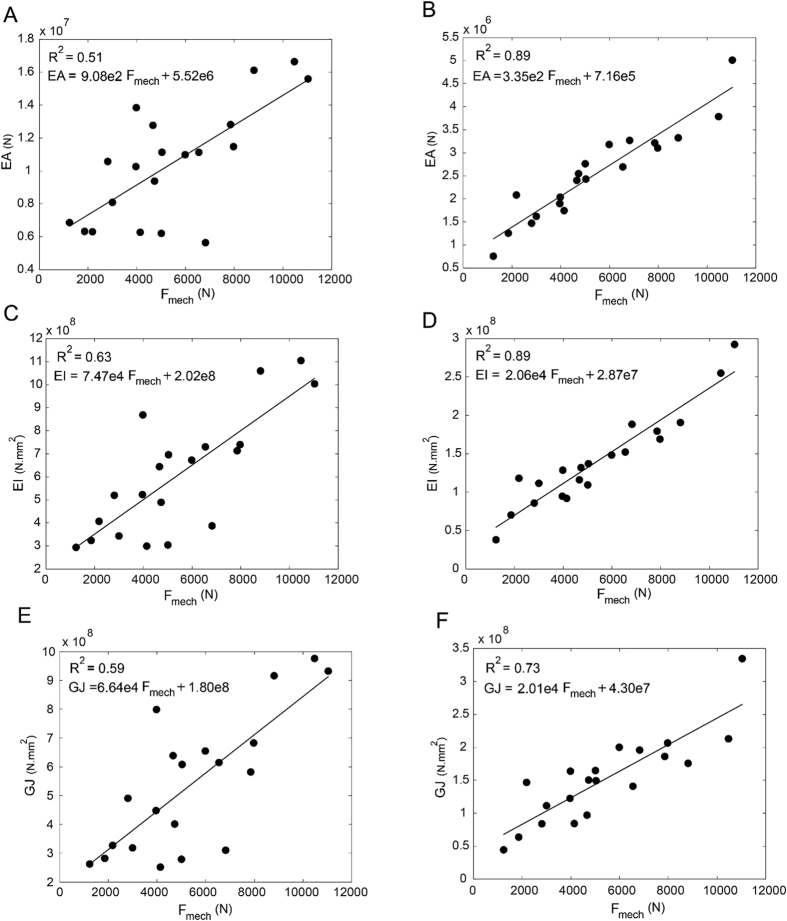
Linear regression between failure load from mechanical testing versus straight beam model (**A**) axial rigidity (**C**) bending rigidity (**E**) torsional rigidity and curved beam (**B**) axial rigidity (**C**) bending rigidity (**F**) torsional rigidity.

When using the best-fit linear regression, rigidity analysis obtained through curved beam CTRA demonstrated a strong correlation with the failure load obtained through mechanical testing (Fig. 5B,D,F). The coefficients of determination for the femurs were 0.89 for *EA* and 0.89 for *EI* and 0.73 for *GJ*, the torsional rigidity which is defined as the sum of *GI*_*x*_ and *GI*_*y*_^12^. For straight beam CTRA, the rigidities are not well correlated with experimental failure load, even when using the best-fit regression, as the coefficients of determination were 0.51 for EA, 0.63 for EI and 0.59 for GJ (Fig. 5A,C,E). Note that Fig. 5C,D are plotted based on minimum bending rigidity (*EI*_*min*_) at each cross-section.

Since mechanical testing, FE analysis and both straight and curved beam CTRA measure failure load on the same specimens, the paired t-test approach has been chosen to assess the mean differences between the pairs. When considering all specimens, paired t-tests did not indicate differences between curved beam CTRA and mechanical testing with an average overestimation of 385 N for failure load (Table 2, P = 0.067). FE analysis demonstrated a mean difference of −197 N compared to mechanical testing, which was not significant (P = 0.24). Straight beam CTRA showed a mean difference of −3315 N when compared to mechanical testing (P < 0.001).

**Figure 6:**
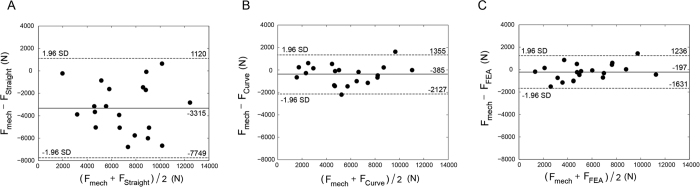
Bland–Altman plots for (**A**) straight beam model (**B**) curved beam model and (**C**) Finite element failure load versus mechanical testing failure load.

The Bland–Altman technique was applied to assess the agreement of straight beam CTRA, curved beam CTRA or FE-based failure load with the gold standard mechanical testing with limits of agreement determined as mean difference ±1.96 standard deviations (i.e., 95% confidence interval of the difference)^31^,^32^. The Bland-Altman Method is based on plotting the difference of two parameters versus their average, in order to analyze the agreement between the two parameters in question. Bland–Altman analysis revealed that the limits of agreement defined as 95% confidence intervals were reasonable for FE and curved beam CTRA models (Fig. 6B,C). For example, the mean difference of −385N for curved beam CTRA model failure load was associated with a precision between −2127 and 1355 N, implying that 95% of the time, curve beam model would provide an estimate of possible difference failure load in the range presented compared to the gold standard mechanical testing. For failure load predicted by curved CTRA (*F*_*Curve*_), the bias was constant across the magnitude of failure load as judged by non-significant correlation between the average versus the difference (r = 0.09, P = 0.71). FE analysis showed more accurate estimates of failure load than each of the two CTRA models (all *P* < 0.001, paired t-tests on the differences versus mechanical testing). The limits of agreement in the Bland–Altman plot indicate that the FE estimated failure load on average is nearly the same as mechanical testing (mean difference of −197 N) and provides estimates that are within the range of −1631 to 1236 N (Fig. 6C, Table 2). Moreover, the bias throughout the magnitude of possible failure loads is constant as indicated by a non-significant correlation between the average versus the difference (r = 0.38, P = 0.10).

To further study differences in prediction of accuracy between the methods, the paired t-tests and Bland–Altman analysis were repeated for the intact and defect specimens separately (Table 2). Paired t-tests then showed a significant difference between mechanical testing and straight beam CTRA for both intact femurs and femurs with lytic defects. The difference between mechanical testing and FE model for the femurs with lytic defects was also significant. In addition, for the curved beam CTRA the limits of agreement varied over the different analyses (total group and both subgroups), but the bias was constant (Table 2). On the other hand, for FE model the limits of agreement were constant for all groups, while the biases were different (Table 2). For the defect femurs, curved beam CTRA showed the smallest bias (−374 N vs. −619 N for FE and −3705 N for straight CTRA), whereas FE showed a higher agreement among predictions (SD 568 N vs. 715 N for curved CTRA and 2218 N for straight CTRA). In addition, correlations between the experiments and the predictions by either FE or curved beam CTRA (all significant at the P = 0.05 level) (Table 2) were found to be high based on the Kendall rank test. This correlation was low for straight beam CTRA for all groups analyzed.

**Figure 7:**
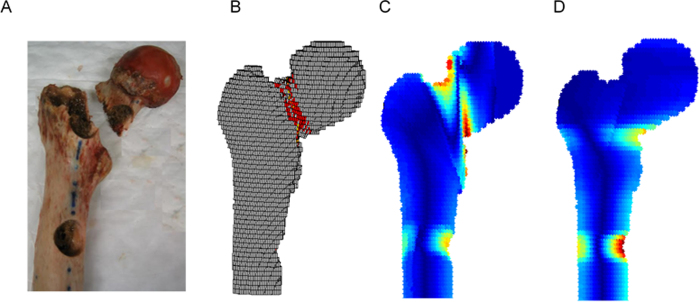
Fracture location as demonstrated by (**A**) mechanical testing, (**B**) Finite element analysis (**C**) Curved beam model and (**D**) straight beam model for a representative sample with defect. For straight and curved beam model the red areas shows the most critical locations which the fracture is imminent.

**Figure 8:**
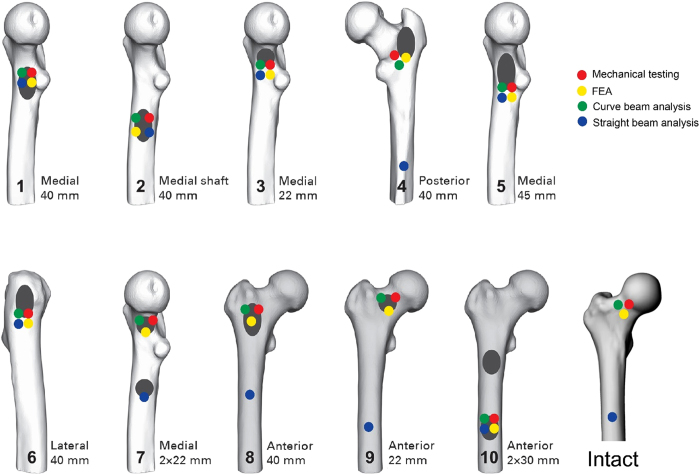
Fracture locations for all specimens as predicted by FEA, curve beam and straight beam models and failure load by mechanical testing.

Fracture locations in the experiments were qualitatively compared to the fracture lines predicted by the FE model and failure location predicated by straight and curved beam CTRA models (Fig. 7 provides a graphic presentation of a representative specimen). The results indicated that the fracture locations were always directed through the lesion in the defect specimens. Overall, the fracture locations were reasonably well predicted by both FE and curve beam model as highlighted in Fig. 8. However, straight beam CTRA was inaccurate in four specimens with lytic defects and in all of the intact specimens.

## Discussion

In recent years, different diagnostic tools have been developed to address the difficulties to predict fracture risk in patients with metastatic bone lesions. The ideal screening test should consider bone as a structure whose mechanical behavior depends on both material and geometric properties. This study evaluated the accuracy of straight and curved beam CTRA models and FE analysis to predict failure load, which was determined through mechanical testing in paired femurs with and without simulated lytic lesions. We were able to demonstrate that predicted failure loads from curved beam CTRA and FE analysis were highly correlated with the actual failure load obtained through mechanical testing. There were no significant differences in prediction accuracy between the two modeling techniques.

The correlation coefficients between the FE analysis predicted and the experimental failure loads (*R*^2^ = 0.91) were similar to those obtained in other FE studies^16^,^21^,^33^,^34^. Similarly, relatively high correlation coefficients between curved beam CTRA and mechanical testing data were evidenced (*R*^2^ = 0.87). However, the correlation between straight beam CTRA and experimental failure load was very poor (*R*^2^ = −0.9) and highly overestimated, since the model was unable to capture the influence of bone curvature in critical cross-sections.

In addition, relatively high correlation coefficients between curved beam CTRA rigidities and mechanical testing data were evidenced (R^2^ = 0.82 and 0.86 for EA and EI respectively). These results are comparable to those obtained by Hong *et al*.^11^, who showed high coefficients of determination when comparing reductions in failure loads versus reductions in axial, bending and torsional rigidity (R^2^ = 0.84, 0.80 and 0.71, respectively) in samples from whale trabecular bone. Similarly, Whealan *et al*.^8^ demonstrated the effectiveness of QCT derived measurements of rigidity for the prospective prediction of yield loads of vertebrae with simulated lytic lesions (*r*_*c*_ = 0.74). Finally, by assessing fracture prediction through benign skeletal lesions in children and young adults, Snyder *et al*.^9^ indicated that bending and torsional rigidities were each highly significant predictors of fracture occurrence and combined, these measures could predict femoral fractures with 97% accuracy. The current results are improvements to previous models^8^,^9^,^11^ by considering rotation in two directions Δ(*dθ*)_*x*_ and Δ(*dθ*)_*y*_ and show better correlation with mechanical testing.

As seen from the rigidity results (Fig. 5), the coefficient of determination for GJ is lower than those of EA and EI. The reason for this lower *R*^2^ can be interpreted as bone structure tends to bend around the axis with lower *EI* in unsymmetrical loading. Therefore, Since the GJ has a linear relationship with minimum and maximum bending rigidity (*GJ* = (*EI*_min_ + *EI*_max_)/(2 + 2*ν*))^12^, the presence of *GI*_max_ in the summation reduces the correlation accuracy for GJ.

In the specimens with a simulated defect, curved beam CTRA seemed to have a higher accuracy (as the bias was lowest), whereas FE analysis showed a higher precision (due to smaller limits of agreement). This could indicate that FE calculations need a correction for bias. In contrast, curved CTRA will provide more accurate estimates of failure load on the group level. However, further studies using larger numbers of specimens are warranted to confirm our findings.

Unlike previously proposed radiographic guidelines, both curved beam CTRA and FE models offer objective assessments of fracture risk by considering the material and geometric properties of bone. Curved beam CTRA is more accurate than straight beam CTRA when predicting both the magnitude of failure loads and the location of the failure. Both techniques are based on QCT imaging, but computational times differ considerably between the two methods. The estimated time for generating and running FE simulations in this study is of 8 hours per sample, and a sophisticated and relatively complex FE software is required to estimate fracture risk. In contrast, curved beam CTRA takes only approximately 15 minutes to estimate fracture risk.

FE simulations are more appropriate for the implementation of complex loading conditions. The decrease in bone strength resulting from metastatic lesions is local and displays a large degree of variability between patients. As a result, forces that insert on the femur close to the lesion site can be more dangerous than larger forces such as the hip contact force. The modelling of such potentially important anatomical characteristics might be more straightforward using FE analysis.

Limitations of our study are shared with many previous works done in the field using *ex-vivo* models for the assessment of failure load prediction using non-invasive imaging methods. Evident differences exist between the metastatic lytic lesions that were artificially simulated in this study and those seen in patients in the clinical practice. In our case, regularly shaped defects were limited to cortical lesions, while metastatic bone lesions generally show an irregular pattern and additionally involve trabecular tissue. However, QCT would be readily able to detect these irregularities and incorporate them into both algorithmic analytical processes, although accurately modeling the material properties of blastic metastatic tissue might be challenging. On a group level, curved beam CTRA and FE analysis accurately predict the femoral load capacity, but on the individual level there can be rather large over- and under-estimations of the femoral strength. These subject-specific over- and under-estimations should be improved before either of the methods can be implemented in clinical practice.

In summary, the results of our study showed that non-invasive subject-specific fracture risk assessment techniques correlate well with actual failure loads measured in mechanical testing experiments. This suggests that curved beam CTRA could be further developed into a tool that can be used in clinical practice. When analyzing the defect femurs only, the results suggested that predictions by FEA are slightly more accurate on a subject-specific level, yet CTRA analysis can be conducted expediently by non-expert operators.

## Additional Information

**How to cite this article**: Oftadeh, R. *et al*. Curved Beam Computed Tomography based Structural Rigidity Analysis of Bones with Simulated Lytic Defect: A Comparative Study with Finite Element Analysis. *Sci. Rep.* **6**, 32397; doi: 10.1038/srep32397 (2016).

## Figures and Tables

**Table 1:** Artificial Lesion sizes and locations on defect femurs.

Specimen	Lesion characteristics	
	Size (mm)	Location
1	40	Med, prox
2	40	Med, shaft
3	22	Med, prox
4	40	Post, prox
5	45	Med, prox
6	40	Lat, prox
7	2 × 22	Med, prox & shaft
8	40	Ant, prox
9	22	Ant, prox
10	2 × 30	Ant, prox & shaft

**Table 2:** Comparison of mechanical testing failure load vs. failure load found from FEA, Curve beam and straight beam model.

Sample No.	Intact				Defect			
	Mech	FEA	Curve	Straight	Mech	FEA	Curve	Straight
1	7852	7416	8533	9318	3002	4149	2856	6163
2	5007	4501	6474	6626	1853	3367	1619	2077
3	5031	5173	5046	10785	2181	2034	2465	7227
4	4728	5197	4192	5587	2806	3541	2201	6462
5	4141	3281	6347	9202	1237	1421	1898	5117
6	4660	4886	4765	8587	3960	4981	5332	10740
7	11034	11477	11047	13861	3980	4976	5419	7134
8	7970	7372	8462	9669	5985	6055	6988	11992
9	6821	7132	7974	13491	6547	7254	6733	11611
10	10470	9029	8852	9827	8815	8787	8594	8899
Failure Load Average (N)	6771 ± 2498	6547 ± 2440	7169 ± 2170	8719 ± 2965	4037 ± 2394	4656 ± 2273	4410 ± 2508	9695 ± 2601
Bland-Altman method, 95% Cl		−1032 to 1482	−2503 to 1707	−7541 to 1693		−1732 to 493	−1776 to 1028	−8052 to 641
Mean ± SD of difference vs. mechanical testing		225 ± 641	−398 ± 1074	−2924 ± 2356		−619 ± 568	−374 ± 715	−3705 ± 2218
P-value (paired t-test)		0.2965	0.2714	0.0035		0.0072	0.1327	0.0005
Kendall Tau ranking coefficients (mechanical testing vs other methods)		0.82	0.73	0.51		0.91	0.87	0.56
	**Total**							
	**Mech**		**FEA**		**Curve**		**Straight**	
Failure Load Average (N)	5404 ± 2764		5601 ± 2491		5790 ± 2685		7742 ± 3111	
Bland-Altman method (N), 95% Cl			−1631 to 1236		−2127 to 1355		−7749 to 1120	
Mean ± SD of difference vs. mechanical testing			−197 ± 731		−385 ± 888		−3315 ± 2262	
P-value (paired t-test)			0.2419		0.067		2.8e-6	
Kendall Tau ranking coefficients (mechanical testing vs other methods)			0.83		0.84		0.53	
